# Trends in Breastfeeding Among Infants Enrolled in the Special Supplemental Nutrition Program for Women, Infants and Children — New York, 2002–2015

**DOI:** 10.15585/mmwr.mm6623a4

**Published:** 2017-06-16

**Authors:** Furrina Lee, Lynn S. Edmunds, Xiao Cong, Jackson P. Sekhobo

**Affiliations:** ^1^Division of Nutrition, New York State Department of Health, Albany, New York; ^2^Department of Epidemiology and Biostatistics, School of Public Health, State University of New York at Albany.

Breastfeeding is widely accepted as the optimal method of infant feeding (*1*,*2*). New York Special Supplemental Nutrition Program for Women, Infants and Children (WIC) has prioritized the promotion of breastfeeding. To assess breastfeeding trends among New York WIC infants, indicators for measuring breastfeeding practices reported by the New York Pediatric Nutrition Surveillance System (PedNSS) during 2002–2015 were examined. The prevalence of breastfeeding initiation increased from 62.0% (2002) to 83.4% (2015), exceeding the *Healthy People 2020* (HP2020)* objective of 81.9% in 2014, with improvements among all racial/ethnic groups. The percentage of New York WIC infants who breastfed for ≥6 and ≥12 months increased from 30.2% and 15.0% (2002) to 39.5% and 22.8% (2015), respectively. The prevalence of exclusive breastfeeding for ≥3 and ≥6 months increased from 8.9% and 2.9% (2006) to 14.3% and 8.0% (2015), respectively. Despite improvements in breastfeeding initiation, increasing the duration of breastfeeding and of exclusive breastfeeding among infants enrolled in the New York WIC program remains challenging. Identifying targeted strategies to support continued and exclusive breastfeeding should remain priorities for the New York WIC program.

The New York WIC administrative data contain records for all participants certified by the program. Race/ethnicity of the infant/child and household income are reported by mothers or caregivers at the time of certification. Answers to questions regarding breastfeeding initiation (“Was [the child] ever breastfed or fed breast milk?”), duration (“How old was [the child] when they stopped being breastfed or fed breast milk?”), and exclusivity (“How old was [the child] when they were first fed something other than breast milk?”) are assessed and updated at each visit until no longer breastfeeding.

New York WIC administrative data are used to generate New York PedNSS files. Non-Hispanic persons are identified as white, black, Asian, or other; persons identified as Hispanic can be of any race. Income is categorized as a percentage of the Federal Poverty Level for a given year. Infants born during the reporting period and who have valid breastfeeding information are included in the breastfeeding initiation analysis. For each category of breastfeeding duration and exclusivity, analyses include only infants who attained the age of interest during the reporting period by their date of visit. During 2002–2015, New York PedNSS reports were used to assess the temporal trends of initiation, duration (i.e., ≥1, ≥3, ≥6, and ≥12 months of breastfeeding), and exclusivity (i.e., ≥1, ≥3, and ≥6 months of exclusive breastfeeding).

Breastfeeding estimates were generated using statistical software.^†^ The National Cancer Institute’s Joinpoint Regression Program 4.2.0.1^§^ was used to test for significance of trends using log-linear transformations for ease of interpretation and comparison, because the models directly provide an estimate of a fixed annual percent change (APC). Statistical significance of trend analysis was defined as p<0.05.

Trend analyses indicated that the racial/ethnic composition of the New York PedNSS cohorts changed during 2002–2015, with significant declines in the percentages of blacks and persons of “other” race/ethnicity (e.g., American Indian or Alaska Native, Native Hawaiian or Other Pacific Islander, multiracial, and unknown), whereas the percentages of Hispanics, whites and Asians increased significantly (Table 1). The percentage of infants enrolled in WIC in New York who were born into families with household incomes ≤100% of the Federal Poverty Level increased significantly from 64.3% in 2002 to 72.9% in 2015 (Table 1).

**TABLE 1:** Number and demographic distribution (i.e., race/ethnicity and poverty status) of infants born during report year (i.e., included in the breastfeeding initiation analysis) — New York Special Supplemental Nutrition Program for Women, Infants and Children, 2002–2015

Year	No. infants	Race/Ethnicity* (%)					Poverty status
		Hispanic	White	Black	Asian	Other^†^	≤100% Federal Poverty Level (%)
2002	122,852	33.0	24.5	26.9	7.5	8.1	64.3
2003	124,436	33.5	25.0	26.2	7.9	7.4	64.9
2004	124,760	34.3	25.2	26.1	8.4	5.9	67.2
2005	105,698	34.7	27.4	26.0	8.9	3.1	64.9
2006	107,385	35.4	26.8	26.0	8.5	3.3	63.9
2007	129,207	36.8	26.1	25.1	9.2	2.8	65.8
2008	131,145	36.5	26.5	25.3	8.8	2.8	66.0
2009	131,550	36.3	26.8	25.3	8.3	3.3	67.7
2010	125,779	36.1	27.4	24.7	8.3	3.5	71.3
2011	126,686	35.7	27.4	24.3	9.2	3.4	73.5
2012	124,622	35.4	27.5	23.9	10.2	3.1	74.9
2013	119,403	35.4	27.8	24.1	9.8	3.0	75.2
2014	117,578	35.7	27.4	23.7	10.2	3.1	74.2
2015	113,806	36.1	26.9	23.5	10.5	3.0	72.9

* Persons identified as Hispanic might be of any race. Persons identified as white, black, Asian, or other race are non-Hispanic. The five racial/ethnic categories are mutually exclusive.

^†^ Includes American Indian or Alaska Native, Native Hawaiian or Other Pacific Islander, multiracial, and unknown.

Breastfeeding initiation among New York WIC infants increased significantly, from 62.0% in 2002 to 83.4% in 2015, with an APC of 2.4 or an average of 1.7 percentage points per year (Table 2). In 2014, the overall prevalence of initiation reached 82.4%, exceeding the HP2020 goal of 81.9%. The HP2020 goal of breastfeeding initiation was reached by Hispanic WIC infants in 2007 (Figure) and has continued to increase by 0.8 percentage points annually. Even larger improvements have been made by other racial/ethnic groups. Asians had the largest relative increase (80.6%) from 45.8% in 2002 to 82.7% in 2015. As of 2015, white infants were also approaching the HP2020 goal for breastfeeding initiation (79.0%). Overall, the racial/ethnic disparity in breastfeeding initiation rate (i.e., the difference between the highest and the lowest rates among white, black, Hispanic and Asian infants in a particular year) was reduced from 26.5 percentage points in 2002 (Hispanic versus Asian) to 9.2 in 2015 (Hispanic versus white).

**TABLE 2:** Percentages of enrolled infants who initiated breastfeeding, continued for ≥1, ≥3, ≥6, or ≥12 months, and who were exclusively breastfed for ≥1, ≥3, or ≥6 months — New York Special Supplemental Nutrition Program for Women, Infants and Children, 2002–2015

Year	Initiation (%)	Duration (%)				Exclusivity (%)*		
		≥1 mon	≥3 mon	≥6 mon	≥12 mon	≥1 mon	≥3 mon	≥6 mon
2002	62.0	56.9	40.6	30.2	15.0	—^†^	—	—
2003	64.6	59.6	43.4	33.1	17.3	—	—	—
2004	66.5	61.5	47.4	38.6	22.7	—	—	—
2005	66.0	60.7	46.9	39.4	25.1	—	—	—
2006	67.2	57.3	42.2	35.5	23.2	15.6	8.9	2.9
2007	72.0	63.6	48.8	39.7	23.4	15.1	8.1	3.3
2008	73.8	64.6	50.0	41.2	26.1	16.4	8.6	3.8
2009	74.4	64.9	49.0	38.8	22.8	17.8	9.7	4.9
2010	76.9	66.9	50.6	38.2	19.7	19.0	10.5	5.8
2011	78.7	68.2	50.5	38.3	20.6	20.4	11.3	6.4
2012	80.1	69.2	51.7	38.0	20.4	20.7	10.9	5.8
2013	81.2	69.3	52.0	39.2	21.4	21.2	11.0	6.3
2014	82.4	70.2	52.9	39.9	22.1	22.1	13.0	7.3
2015	83.4	71.7	53.9	39.5	22.8	23.2	14.3	8.0

* Breastfeeding exclusivity information was not collected by New York State Special Supplemental Nutrition Program for Women, Infants and Children before 2006.

^†^ Data not available for these years.

**FIGURE:**
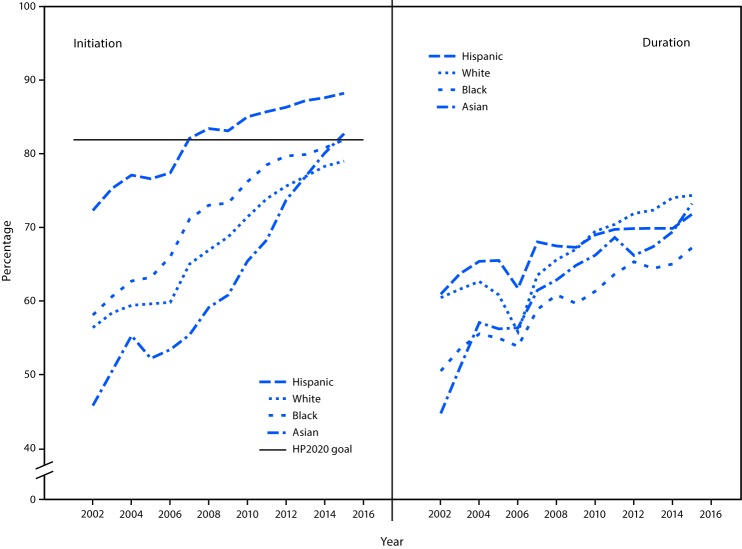
Breastfeeding initiation and duration for ≥1 month among Hispanic, white, black, and Asian infants* — New York Special Supplemental Nutrition Program for Women, Infants and Children, 2002–2015 **Abbreviation:** HP2020 = *Healthy People 2020*. * Persons identified as Hispanic might be of any race. Persons identified as white, black, Asian, or other race are non-Hispanic. The five racial/ethnic categories are mutually exclusive.

There was a significant increase in the crude prevalence of breastfeeding duration during 2002–2015 for infants who breastfed for ≥1 month (APC = 1.7) and ≥3 months (APC = 1.9) (Table 2). Joinpoint regression analysis of breastfeeding prevalence for ≥6 months indicated two segments. During 2002–2004 (APC = 13.2), the increase was not significant at the p<0.05 level but would have been at the 0.06 level; and during 2004–2005 (APC = 0.3), the prevalence leveled off. Similarly, the percentage of infants who breastfed for ≥12 months increased from 2002–2005 (APC = 17.2), and then leveled off from 2005–2015 (p = 0.90). Further examination of all breastfeeding duration trends by race/ethnicity demonstrated significant improvements among all racial/ethnic groups only for breastfeeding duration of ≥1 month, with the largest increase occurring among Asians (Figure). Overall, 71.7% of these infants were breastfed for ≥ 1 month in 2015.

Exclusive breastfeeding status among WIC infants was not monitored by PedNSS until 2006 (Table 2). During 2006–2015, exclusive breastfeeding (≥1 and ≥3 months) increased significantly, with an APC of 5.0 and 5.8, respectively. The percentage of infants exclusively breastfed for ≥6 months increased significantly during 2006–2010 (APC = 18.9), and again during 2010–2015 (APC = 6.2).

## Discussion

The New York WIC program reached the HP2020 breastfeeding initiation goal of 81.9% 6 years ahead of target, with substantial increases in all racial/ethnic groups during 2002–2015. Despite considerable progress in breastfeeding duration over time, the New York WIC program was still 21.1 percentage points below the HP2020 objectives for breastfeeding duration ≥6 months (60.6%) and 11.3 percentage points below the HP2020 objectives for breastfeeding duration ≥12 months (34.1%) in 2015. The crude prevalence of exclusive breastfeeding for ≥3 months (14.3%) and ≥6 months (8.0%) in 2015 were less than one-third of the 46.2% and 25.5% HP2020 objectives, respectively. If the current pace continues, the New York WIC program will not achieve the HP2020 goals for duration and exclusivity during the next 5 years.

At the national level, the U.S. Department of Agriculture and the U.S. Department of Health and Human Services have led efforts to promote breastfeeding through signature initiatives (e.g., the *Loving Support* program,**^¶^** the *Surgeon General’s Call to Action to Support Breastfeeding*,** and the *Healthy People* objectives.) The New York WIC program has a long history of promoting breastfeeding as a strategy to prevent childhood obesity.^††^ Moreover, New York and local governments enacted legislation (*3*) and the *Latch-On*^§§^ initiative to improve support of breastfeeding. The substantial progress in breastfeeding measures among New York WIC infants likely reflects the collective efforts at national, state and local levels. However, the observed trends indicate that among New York WIC infants these efforts might be more effective at improving initiation rather than duration and exclusivity, and that breastfeeding practices might vary by race or ethnicity.

WIC provides multiple services (including supplemental foods when applicable) to all infants, children, and mothers enrolled in the program. In 2009, the economic value of the food packages issued to fully breastfeeding mothers was enhanced (*4*). However, a recent study, using 2004–2010 data from multiple sources, demonstrated little effect of these changes on various breastfeeding measures (*5*). The analyses presented here, with an additional 5 years of New York PedNSS data, support those findings. In particular, the annual increase in breastfeeding initiation remains steady among different racial/ethnic groups. Joinpoint regression analyses of breastfeeding duration and exclusivity trends showed no inflection point at 2009 (or 2010 if there was a delayed response), suggesting little or no association with the 2009 food package changes as well.

The trends of breastfeeding initiation (2004–2011) and duration (≥4 weeks, 2004–2011) illustrated by the New York PedNSS are similar to those among “on-WIC during pregnancy”–participants residing in New York reported by the Pregnancy Risk Assessment Monitoring System^¶¶^ (PRAMS). In addition to a higher response rate and shorter recall interval, timely dissemination of breastfeeding statistics of infants living in low income households and participating in WIC is one advantage of the PedNSS over the PRAMS. This is of particular importance, because a prompt program evaluation is an integral part in the adaptive and iterative design of any quality improvement project. Nevertheless, these two surveillance systems were developed with distinct objectives and thus collect data from different sources. The complementary information provided by the PedNSS and the PRAMS strengthens the surveillance efforts related to improving infant health.

The findings in this report are subject to at least two limitations. First, the observed improvements in breastfeeding outcomes could not be attributed to any particular exposure(s) (e.g., a specific breastfeeding promotion initiative). Second, because this analysis was conducted among New York WIC participants, the findings might not be generalizable to populations enrolled in other programs or in other parts of the country.

The decision to continue breastfeeding is influenced by a combination of demographic, socioeconomic, psychosocial, cultural, and environmental factors (*3*,*6*–*8*). The findings in this study indicate potential conceptual or methodological limitations in existing initiatives to promote duration and exclusivity. The challenge for the New York WIC program, which might be applicable to WIC programs in other states, is to identify those elements that might be influential to a majority of mothers in low-income households regarding breastfeeding duration and exclusivity from participants’ perspectives; design theory-based interventions that optimize the existing resources available in the program itself, communities, and the health care system (*9*); implement the interventions with high fidelity (i.e., measured and assessed in terms of adherence and competence) (*10*); and evaluate the efficacy of the interventions regularly using mixed-method approaches.

**Summary**

**What is already known about this topic?**

Breastfeeding is widely accepted as the optimal method of infant feeding. Collective efforts at national, state, and local levels have been made to promote breastfeeding initiation, duration and exclusivity among low-income families.

**What is added by this report?**

Breastfeeding initiation among New York infants enrolled in the Special Supplemental Nutrition Program for Women, Infants and Children (WIC) exceeded the 81.9% *Healthy People 2020* *(*HP2020) objective in 2014. The racial/ethnic disparity in initiation declined from 26.5 percentage points in 2002 to 9.2 in 2015. Although significant progress has been made regarding breastfeeding duration and exclusivity (e.g., 39.5% breastfeeding for ≥6 months and 14.3% exclusively breastfeeding for ≥3 months in 2015, respectively), the New York WIC program is not on target to meet the HP2020 objectives of 60.6% (≥6 months duration) and 46.2% (≥3 months exclusively), respectively. Improvements in breastfeeding measures vary by race/ethnicity.

**What are the implications for public health practice?**

Current interventions are effective in promoting breastfeeding initiation and helpful in improving duration of breastfeeding among some racial/ethnic groups of New York WIC participants. In addition to known best practices, future breastfeeding promotion strategies should explore these limitations and focus on implementation with high fidelity.

## Footnotes

*
https://www.healthypeople.gov/2020/About-Healthy-People .

† SAS Institute Inc., Cary, NC.

§ Statistical Research and Applications Branch, National Cancer Institute, May 2015.

¶
https://lovingsupport.fns.usda.gov/ .

**
https://www.surgeongeneral.gov/library/calls/breastfeeding/index.html .

††
https://www.health.ny.gov/prevention/nutrition/resources/eat_well_play_hard/ .

§§
https://www1.nyc.gov/site/doh/health/health-topics/breastfeeding-latch-on.page .

¶¶
https://www.health.ny.gov/statistics/prams/ .
